# Integrating Single-Step GWAS and Bipartite Networks Reconstruction Provides Novel Insights into Yearling Weight and Carcass Traits in Hanwoo Beef Cattle

**DOI:** 10.3390/ani10101836

**Published:** 2020-10-09

**Authors:** Masoumeh Naserkheil, Abolfazl Bahrami, Deukhwan Lee, Hossein Mehrban

**Affiliations:** 1 Department of Animal Science, University College of Agriculture and Natural Resources, University of Tehran, Karaj 77871-31587, Iran; Naserkheil@ut.ac.ir (M.N.); a.bahrami@ut.ac.ir (A.B.); 2 Department of Animal Life and Environment Sciences, Hankyong National University, Jungang-ro 327, Anseong-si, Gyeonggi-do 17579, Korea; 3 Department of Animal Science, Shahrekord University, Shahrekord 88186-34141, Iran; hosseinmehrban@gmail.com

**Keywords:** weighted single-step, GWAS, candidate gene, biological pathways, Hanwoo

## Abstract

**Simple Summary:**

Hanwoo is an indigenous cattle breed in Korea and popular for meat production owing to its rapid growth and high-quality meat. Its yearling weight and carcass traits (backfat thickness, carcass weight, eye muscle area, and marbling score) are economically important for the selection of young and proven bulls. In recent decades, the advent of high throughput genotyping technologies has made it possible to perform genome-wide association studies (GWAS) for the detection of genomic regions associated with traits of economic interest in different species. In this study, we conducted a weighted single-step genome-wide association study which combines all genotypes, phenotypes and pedigree data in one step (ssGBLUP). It allows for the use of all SNPs simultaneously along with all phenotypes from genotyped and ungenotyped animals. Our results revealed 33 relevant genomic regions related to the traits of interest. Gene set enrichment analysis indicated that the identified candidate genes were related to biological processes and functional terms that were involved in growth and lipid metabolism. In conclusion, these results suggest that the incorporation of GWAS results and network analysis can help us to better understand the genetic bases underlying growth and carcass traits.

**Abstract:**

In recent years, studies on the biological mechanisms underlying complex traits have been facilitated by innovations in high-throughput genotyping technology. We conducted a weighted single-step genome-wide association study (WssGWAS) to evaluate backfat thickness, carcass weight, eye muscle area, marbling score, and yearling weight in a cohort of 1540 Hanwoo beef cattle using BovineSNP50 BeadChip. The WssGWAS uncovered thirty-three genomic regions that explained more than 1% of the additive genetic variance, mostly located on chromosomes 6 and 14. Among the identified window regions, seven quantitative trait loci (QTL) had pleiotropic effects and twenty-six QTL were trait-specific. Significant pathways implicated in the measured traits through Gene Ontology (GO) term enrichment analysis included the following: lipid biosynthetic process, regulation of lipid metabolic process, transport or localization of lipid, regulation of growth, developmental growth, and multicellular organism growth. Integration of GWAS results of the studied traits with pathway and network analyses facilitated the exploration of the respective candidate genes involved in several biological functions, particularly lipid and growth metabolism. This study provides novel insight into the genetic bases underlying complex traits and could be useful in developing breeding schemes aimed at improving growth and carcass traits in Hanwoo beef cattle.

## 1. Introduction

To date, demand for high-quality animal protein is increasing, bringing beef quality and consumer satisfaction to the spotlight within the beef industry. Hanwoo cattle, which is unique to Korea, is characterized by bountiful marbling of meat and quality attributes, such as tenderness, juiciness, and good flavor [1]. The main aim of the Hanwoo beef industry is to increase the quantity and improve the quality of the meat. To achieve this goal, estimated breeding values (EBVs) for carcass weight (CW), backfat thickness (BT), eye muscle area (EMA), marbling score (MS), and yearling weight (YW) are commonly included in the selection criteria of breeding programs [2]. Despite the importance of genetic improvement for more targeted meat production in the Hanwoo cattle industry, little is known about the underlying genomic architecture influencing these invaluable traits. In recent decades, the advent of high throughput genotyping technologies has made it possible to perform a genome-wide association study (GWAS) for the detection and localization of genomic regions associated with traits of economic interest in different species [3]. Recent studies in beef cattle for growth and carcass traits have revealed major quantitative trait loci (QTL) on chromosomes 6, 8, 12, 14 and 20 using a GWAS approach [4,5,6]. However, more studies are needed to provide further insight into the chromosomal regions, causal markers and candidate genes that affect related traits [7].

Among the approaches used for GWAS, the classical method is based on testing a single marker at a time as a fixed effect in a single-locus mixed linear model, which includes the pedigree or genomic relationship matrix to infer QTL from single SNP effects [8]. The main benefit of classical GWAS (CGWAS) is the ease of significance testing; however, some of the challenges of this method include false positives and the overestimation of QTL effects [9]. Consequently, it is likely to result in a reduced fit of the data compared to methods that all SNPs are considered together [10]. Therefore, Bayesian approaches were developed as they offer methods to mitigate these challenges [11], although this increases computation demand. Nevertheless, in both single-marker and Bayesian procedures; only animals with known phenotype and genotype are included in the analysis. On the other hand, Wang et al. [10] proposed an alternative approach for GWAS, termed the weighted single-step GWAS (WssGWAS), which combines all genotypes, phenotypes and pedigree data in one step (ssGBLUP). It allows for the use of all SNPs simultaneously along with all phenotypes from genotyped and ungenotyped animals. This approach is accomplished by the conversion of estimated breeding values (GEBVs) to marker effects and weights, which are then used in an iterative process to update the SNP solutions and improve the statistical power of QTL detection [10,12]. Furthermore, this method with marker weights is faster, more accurate and simpler to implement for GWAS applications than other multistep approaches [10]. Several recent studies have used a weighted single-step procedure to detect effective QTL on production, carcass and reproductive traits in different species [13,14,15,16]. The integration of GWAS results in quantitative traits with pathway and gene-set enrichment analyses in dairy cattle has recently gained interest [17,18,19]. Complex traits are products of a series of multi-omics layers, including genomics, epigenomics, transcriptomics, proteomics, and metabolomics. Incorporating GWAS data with omics layers data has powerfully detected biological mechanisms of traits [20,21,22]. While the previous systems biology approach frequently focused on integration with transcriptomics data [23], to further elucidate the complicated biology of the traits, the innovative integration of layers of omics has been endorsed. Moreover, further insight into gene sets and biological pathways that regulate related bovine traits might help to detect genes associated with growth and carcass traits. Systems biology approaches and bipartite network analyses have mostly been used as complementary approaches for extracting biological information from omics layers and increased our understanding of the complex traits [17].

Several genes associated with carcass traits in Hanwoo beef cattle were identified in the literature using either a single marker or the Bayesian model [24,25,26,27,28]; however, these studies considered neither WssGWAS nor the detection of genomic regions associated with YW, which is an important trait to identify when selecting young bulls [2]. Therefore, the purposes of this study were to perform GWAS using a weighted single-step approach to detect genomic regions and candidate genes associated with yearling weight and carcass traits in Hanwoo cattle and compare the results with previous studies. In addition, harnessing this enormous work, here we extended a new viewpoint to appraise the enrichment of WssGWAS data with miRNA-gene network reconstruction to further detect the contribution of miRNA function in each trait. This integration enables us to survey post-transcriptional regulation and also detect candidate genes and miRNAs that are essential in growth and carcass traits.

## 2. Materials and Methods

### 2.1. Ethics Statement

Semen and blood samples for genotyping were provided by the Hanwoo Improvement Center of the National Agricultural Cooperative Federation. Ethics committee approval was not required for semen and blood collection, as they were collected specifically in this study from the Hanwoo Improvement Center (HIC), which was involved as a partner in this research project, which was supported by a grant from the IPET Program (No. 20093068), Ministry of Agriculture, Food and Rural Affairs, Republic of Korea. Pedigree data were recorded by the Korean Animal Registration Association, data for growth traits were obtained from the Hanwoo Improvement Center, and data for carcass traits were recorded by special inspectors, from the Institute of Korean Animal Products Evaluation, at the slaughterhouse through the progeny testing program in Korea. Pedigree and phenotypic data related to growth and carcass traits were generated following the protocol for the progeny test program, as notified by the Ministry of Agriculture, Food and Rural Affairs, based on livestock law in Korea. The Hanwoo Improvement Center, as an enforcement institution for the testing program for selecting proven Hanwoo bulls, is obligated to maintain data and ownership of enrolled animals under notice.

### 2.2. Phenotypic and Pedigree Data

The data were obtained from phenotypes recorded of 15,279 animals (8966 bulls and 6313 steers) for yearling weight, of which 5824 steers with carcass traits were provided by the Hanwoo Improvement Center of the National Agricultural Cooperative Federation. The steers were born from 1989 to 2015 and castrated at 5 months of age. The pedigree information of 50,115 animals was used in the animal model after tracing back the pedigree file to 11 previous generations. The phenotypic records were available for backfat thickness (BT), carcass weight (CW), eye muscle area (EMA), marbling score (MS), and yearling weight (YW) traits (Table 1). Marbling score was assessed using a categorical system of nine classes ranging from the lowest score of one (no marbling) to the highest of nine (abundant marbling). The collected records of MS before 2005 were removed because of mismatches with the newly adopted 1-to-9-point marbling score scale. Carcass traits were measured according to the Korean carcass grading procedure of the steers at—approximately 24 months of age, ribbed between the 13th rib and the first lumbar vertebrae after 24 h postmortem—and by notification No. 2014-4 of the Ministry of Agriculture, Food, and Rural Affairs. YW trait for each animal was determined from the weight (W_t_) at the termination (t) of the test (body weight at ~12 months) and the previous weight (W_t−1_) recorded at a time point (t_-1_) before (body weight at ~6 months) the termination, according to the equation described by Park et al. [29]:

$$\mathrm{YW}=\left[\left(\frac{W_{t}-{\;W}_{t_{-1}}}{t\;-{\;t}_{-1}}\right)\times \left(365-{\;t}_{-1}\right)\right]+{\;W}_{t_{-1}}$$

### 2.3. Genotypic Data

The genotyping for 1679 animals was performed using the Illumina BovineSNP50K BeadChip (Illumina Inc. San Diego, CA, USA). Animals with more than 10% missing data (*n* = 73), parent–progeny conflicts (*n* = 11) and deviation error between pedigree and genomic relationships (n = 39), in addition to a lack of phenotypic record for five traits (*n* = 16), were removed from the final analyses. Out of 45,304 SNPs, the quality control of genomic data excluded the SNPs with an unknown position (302 SNPs) and those located on sex chromosomes (1150 SNPs). Additionally, the SNPs with call rates lower than 0.98 (2677 SNPs), minor allele frequencies lower than 0.01 (6684 SNPs), and a maximum difference between the observed and expected frequency of 0.15 as the departure of heterozygous from Hardy–Weinberg equilibrium (31 SNPs) were excluded from the analyses [30,31]. In the final analysis, genotypes on 34,460 SNP markers from 1540 animals (385 bulls and 1155 steers) were available. All genotyped animals had records for YW, whereas four genotyped steers lacked records for carcass traits. Consequently, the number of animals in the genotyped files for carcass traits and YW were 1151 and 1540, respectively.

### 2.4. Analysis of the Single-Step Genomic Association

The WssGBLUP was performed using BLUPF90 family software [32]. First, variance components were determined using the pedigree-based univariate model obtained by Mehrban et al. [31]. A single-trait animal model to predict genomic breeding values was as follows:

y=Xb+Za+e

where, **y** is the vector of observations; **b** is the vector of fixed effects including batch-test place-sex (163 levels) and birthplace (108 levels) for YW trait; batch-test place-slaughter date (391 levels, 201 levels for MS), birth place (86 levels, 76 levels for MS) and slaughter age as a covariate for carcass traits (except BT, as there is no significant effect of slaughter age); **a** is the vector of random animal effects assumed to follow N (**0**, **H**$\sigma_{a}^{2}$), with **H** representing the additive relationship matrix that combines pedigree (**A**) and genomic information (**G**); $\sigma_{a}^{2}$ is the additive genetic variance; **e** is the vector of random residual effects that the distribution is assumed to follow N (**0**, **I**$\sigma_{e}^{2}$), where **I** is an identity matrix including all animals with records and $\sigma_{e}^{2}$ is the residual variance; **X** and **Z** are incidence matrices that relate records to fixed effects and random animal effects, respectively.

The matrix **H**^−1^ was constructed [33] as follows using PREGSF90 program [34]:

$$H^{-1}=A^{-1}+\;\left[\begin{array}{cc} 0 & 0\\ 0 & {\left(0.95G+0.05A_{22}\right)}^{-1}-A_{22}^{-1} \end{array}\right]$$

where **A** is a numerator relationship matrix; **G** is the genomic relationship matrix; **A**_22_ is the numerator relationship matrix for genotyped animals. To avoid singularity problems, the coefficients of **G** and **A**_22_ were considered 0.95 and 0.05, respectively [35].

The **G**-matrix was built using the information of genome-wide dense markers [35]:

$$G=\frac{MDM^{\prime }}{2\sum_{i=1}^{m}p_{i}\;\left(1-p_{i}\right)},$$

where m is the total number of markers (34,460), p_i_ is the allelic frequency of ith marker, **M** is the matrix of centered genotypes and **D** is a diagonal matrix of weights for markers (initially **D** = **I**). According to Zhang et al. [12], the following steps obtained estimates of SNP effect and weights for WssGWAS:

In the first iteration (t = 1) **D** = **I**, $G_{\left(t\right)}=\;\frac{MD_{\left(t\right)}M^{\prime }}{2\sum_{i=1}^{m}p_{i}\;\left(1-p_{i}\right)}$;

Predict GEBV ($\hat{a}$) using ssGBLUP approach;

Markers effect ($\hat{u}$) was estimated from GEBVs ($\hat{a}$) as ${\hat{u}}_{\left(t\right)}=D_{\left(t\right)}M^{\prime }G_{\left(t\right)}^{-1}{\hat{a}}_{\left(t\right)}$;

Calculate the common weight for a group of 20 adjacent SNP as $\sum_{i=1}^{20}{\hat{u}}_{i}^{2}$;

Normalize SNP weights to keep the total genetic variance constant:

$$D_{\left(t+1\right)}=\frac{tr\left(D_{\left(t\right)}\right)}{tr\left(D_{\left(t+1\right)}\right)}D_{\left(t+1\right)};$$

Reconstruct $G_{\left(t+1\right)}=\;\frac{MD_{\left(t+1\right)}M^{\prime }}{2\sum_{i=1}^{m}p_{i}\;\left(1-p_{i}\right)}$;

t = t + 1 and loop to step 2.

Lourenco et al. [36] indicated that direct genomic values (DGV) are a more relevant starting point than GEBV because animals with different levels of accuracy may be included in genotyped populations. Hence, GEBV ($\hat{a}$) was replaced by DGV in this stage, which was obtained as follows [33]:

$${\mathrm{DGV}}_{i}=-(\underset{j\neq i}{\sum }g^{\mathrm{ij}}{\;\mathrm{GEBV}}_{j}/g^{\mathrm{ii}})$$

where g^ij^ is the element in **G**^−1^ corresponding to relationships between animal i and j.

Updating SNP weights were continued for only two iterations in each trait because of the decreasing accuracy of genomic breeding values in the succeeding iterations. According to Wang et al. [15], the explained genetic variance of adjacent 20 SNPs on the genome was obtained as

$$\frac{var(\sum_{i=1}^{20}Z_{i}{\hat{u}}_{i})}{\sigma_{a}^{2}}\times 100.$$

### 2.5. Selection of Relevant SNP Windows and Putative Candidate Genes Identification

Consecutive 20 SNPs, which explained 1% or more of the total genetic variance, based on the WssGWAS, were considered as a genomic window associated with the studied traits [37,38,39]. The Map Viewer tool for the bovine genome was used to identify positional candidate genes based on the starting and ending coordinates of each window using the UMD 3.1.1 assembly as the reference map (https://www.ncbi.nlm.nih.gov/genome/gdv/?org=bos-taurus). In order to understand the action of these genes on the traits investigated the biological function of annotated genes were explored by GeneCards (www.genecards.org). A Manhattan plot was created using the R software [40].

### 2.6. Functional Gene Set Annotation and Enrichment

Gene set annotation and enrichment analysis was performed using online programs, DAVID (the Database for Annotation, Visualization, and Integrated Discovery) [41], PANTHER (Protein ANalysis THrough Evolutionary Relationships) [42], version 14, and g:Profiler [43], comprehensive web tools for scholars and researchers to understand the biological meaning of multiple genes and gene ontology (GO) terms.

### 2.7. Target Gene Prediction and Validation of Candidate miRNAs

The potentially targeted genes were predicted using miRWalk [44]. The platform integrates information from different miRNA-target databases, including validated information and prediction datasets: MiRWalk, miRDB, miRMap, miRNAMap, MicroT4, miRanda, miRBridge, PICTAR2, RNAhybrid, Targetscan, PITA and RNA22. The target genes that were predicted by at least five mentioned tools were chosen and submitted to DAVID, KEGG (the potential Kyoto Encyclopedia of Genes and Genomes) [45,46], Reactome pathways and PANTHER databases for the enrichment target genes of each miRNA.

### 2.8. Gene-Traits and miRNA–Gene Network Reconstruction

Gene-traits network was manually reconstructed based on traits as source nodes and genes as target nodes in Cytoscape software. The miRNA–gene network was reconstructed based on the candidate genes and the molecular interactions documented in related papers and online interaction databases. Protein–protein interaction (PPI) data were abstracted from the Biomolecular Interaction Network Database (BIND) [47], Database of Interacting Proteins (DIP) [48], Biological General Repository for Interaction Datasets (BioGRID) [49], and Mammalian Protein–Protein Interactions Database (MIPS) [50]. In addition, pathway data were obtained from searches in pathway databases, such as STRING (Search Tool for the Retrieval of Interacting Genes/Proteins) [51]. Each gene and miRNA was entered into the database, and resulting interactions were imported to the networks using Cytoscape 3.7.2 (National Institute of General Medical Sciences, Bethesda Softworks, Rockville, MD, USA) Cytoscape plugins were done for analyzing unified interactive data. Genes and miRNAs in generated networks are represented as nodes and the interactions between these nodes as edges.

## 3. Results

### 3.1. Summary Statistics

The five traits selected for GWAS analysis were backfat thickness (BT), carcass weight (CW), eye muscle area (EMA) and marbling score (MS), and yearling weight (YW). The descriptive statistics of these traits are provided in Table 1.

### 3.2. Association Analysis

**Figure 1:**
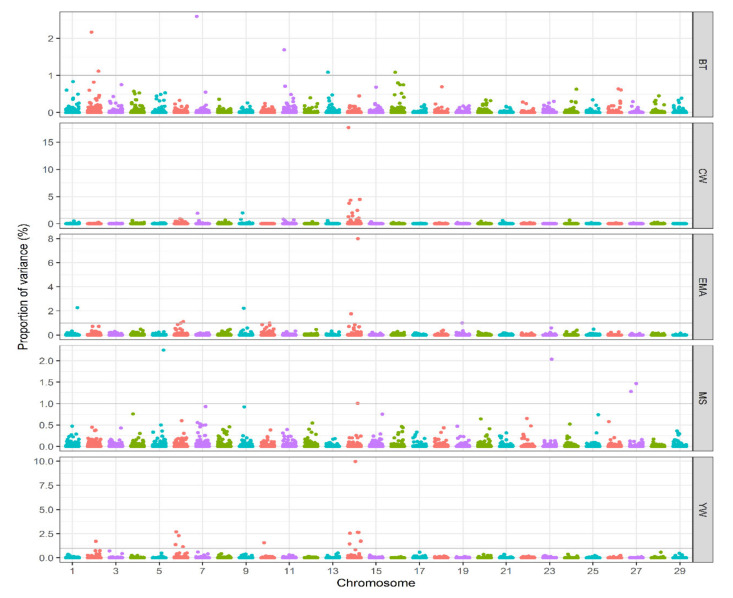
Manhattan plots of the percentage of additive genetic variance explained by windows of 20 adjacent SNPs for all studied traits using R software (version 4.0.2). BT, Backfat Thickness; CW, Carcass Weight; EMA, Eye Muscle Area; MS, Marbling Score; YW, Yearling Weight.

In this study, we identified genomic regions associated with five measured traits in Hanwoo cattle using WssGWAS. Manhattan plots showing the proportion of genetic variance explained by the window of 20 adjacent SNPs (~ 1.39 Mb) for the traits under study are in Figure 1.

A summary of each SNP window that explained more than 1% of additive genetic variance and positional candidate genes is presented in Table 2. Only genes inside the significant windows were identified for yearling weight and carcass traits. A total of thirty-three relevant genomic regions were found to be associated with considered traits in our study. Furthermore, the identified significant windows explained totally 9.73, 42.31, 17.35, 8.03, and 33.44% of genetic variances for BT, CW, EMA, MS, and YW, respectively. Six genomic regions on chromosomes 2, 7, 11, 13 and 16 for BT and eleven genomic regions on chromosomes 7, 9 and 14 for CW were located. The largest QTL window that explained 17.66% of genetic variance for CW was located in the region of 24.58–25.33 Mb on chromosome 14. Analyses for EMA identified seven genomic regions as the most important loci; they were distributed on chromosomes 1, 6, 9, 14 and 19, which together accounted for 17.35% of the additive genetic variance. The same windows observed for EMA on chromosome 14 in the regions of 22.09–23.61 Mb and 24.58–25.33 Mb appeared to also influence CW. Five genomic regions that explain more than 1% of the additive genetic variance were observed on chromosomes 5, 14, 23 and 27 for MS. There were thirteen genomic regions associated with YW on chromosomes 2, 6, 10 and 14. Of these significant windows, four windows were located on chromosome 6, explaining 7.50% of the total variance and seven windows were on chromosome 14, which explains 22.66% of total variance for YW. Significant QTL windows that were associated with more than one trait were defined as pleiotropic QTL. The results indicated that ten large-effect windows located on chromosome 14 are likely to be involved in one or more traits, in which seven QTL were common and the other three were trait-specific. Using the Bos taurus genome map, numerous candidate genes within the significant regions (SNP window)—some of which have been previously reported to be associated with the traits of interest (BT, CW, EMA, MS, and YW) in several cattle breeds—were identified in our study. A total of 371 genes were annotated in these genomic regions according to the National Center for Biotechnology Information (NCBI), which along with general information about results of WssGWAS for the analyzed traits are presented in Table 2.

### 3.3. Functional Gene Set Annotation and Enrichment

**Figure 2:**
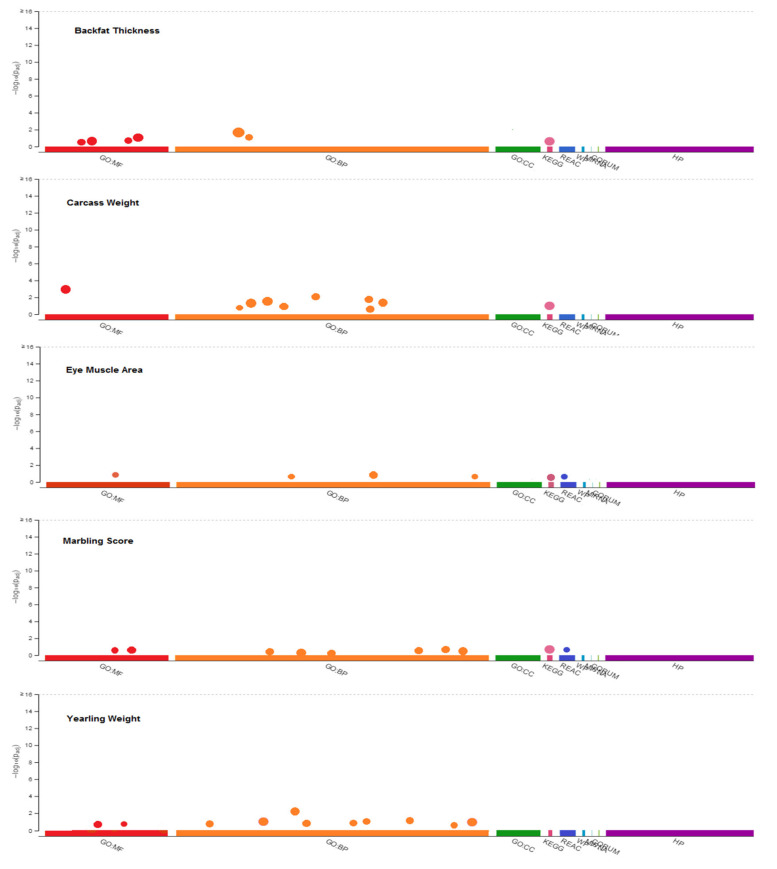
Functional enrichment analysis or gene set enrichment analysis, on input gene list for related traits using g: Profiler (https://biit.cs.ut.ee/gprofiler). MF: Molecular Functions, BP: Biological Process, CC: Cellular Component, KEGG: Kyoto Encyclopedia of Genes and Genomes, REAC: Reactome pathways, WP: WikiPathways, MIRNA: MicroRNAs, CORUM: comprehensive resource of mammalian protein complexes and HP: Human Phenotype Ontology.

GO terms functional annotation was first performed to identify the biological meaning and the systematic features of these candidate genes using DAVID, PANTHER, and g:Profiler databases. The gene ontology contains nine categories: Molecular Functions (MF), Biological Process (BP), Cellular Component (CC), Kyoto Encyclopedia of Genes and Genomes (KEGG), Reactome pathways (REAC), WikiPathways (WP), MicroRNAs (MIRNA), comprehensive resource of mammalian protein complexes (CORUM) and Human Phenotype Ontology (HP). The significantly different GO terms of candidate genes for each trait are presented in Figure 2 (*p* < 0.05), including the 6, 9, 4, 8 and 11 Go terms related to BT, CW, EMA, MS, and YW, respectively. Pathway enrichment analysis revealed that the genes involved in BT, CW, EMA, MS, and YW were enriched in the Rap1 signaling pathway, Neuroactive ligand–receptor interaction, Cell adhesion molecules and PECAM1 interactions, and MAPK signaling pathway and RAF/MAP kinase cascade, respectively. Functional gene set annotation and enrichment were presented in Table 3.

### 3.4. Prediction of miRNA-Target Genes

Among the detected regions, three miRNAs were identified: bta-miR-124a, bta-miR-3064, and bta-miR-2379. The identified miRNAs suppressed 42 of the candidate genes, including ABT1, ATF7IP, ATP6V1H, B3GNT2, C14H8orf46, C19H17orf58, CCDC115, CDKN1B, CGA, CLASP1, DERL1, EXO1, FAM135B, FAM234B, FAM83A, FAM91A1, GRK7, HEBP1, NIFK, NIPA1, NKAIN3, NSMCE2, PPARGC1A, PPM1L, PPP1R42, PRELID1, PRELID2, PXYLP1, RALGAPA2, RNF111, RNF7, SGK3, SLC31A1, SLTM, SMURF2, ST18, STK32A, TFDP2, TMEM68, TRIM55, ZHX1 and ZNF346 for all traits.

### 3.5. Gene-Traits and miRNA–Gene Network Reconstruction

**Figure 3:**
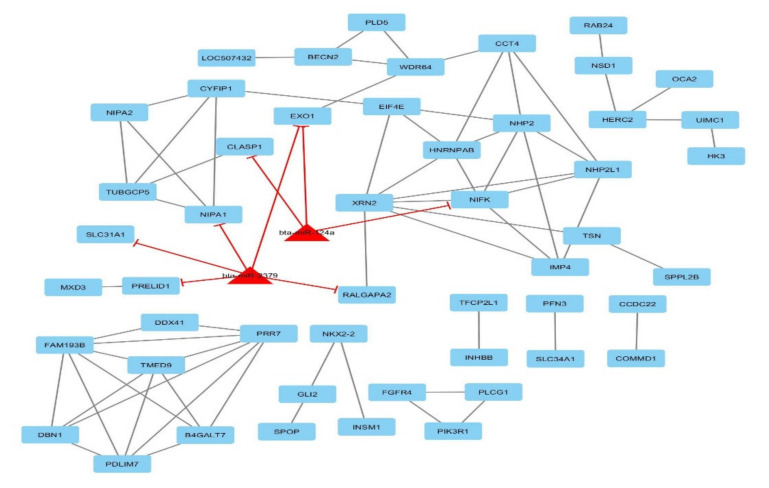
The network of backfat thickness related genes and miRNAs using Cytoscape software (version 3.7.2; www.cytoscape.org); each node denotes a gene (rectangle) and miRNA (triangle) and edges depict miRNA–gene relationships.

**Figure 4:**
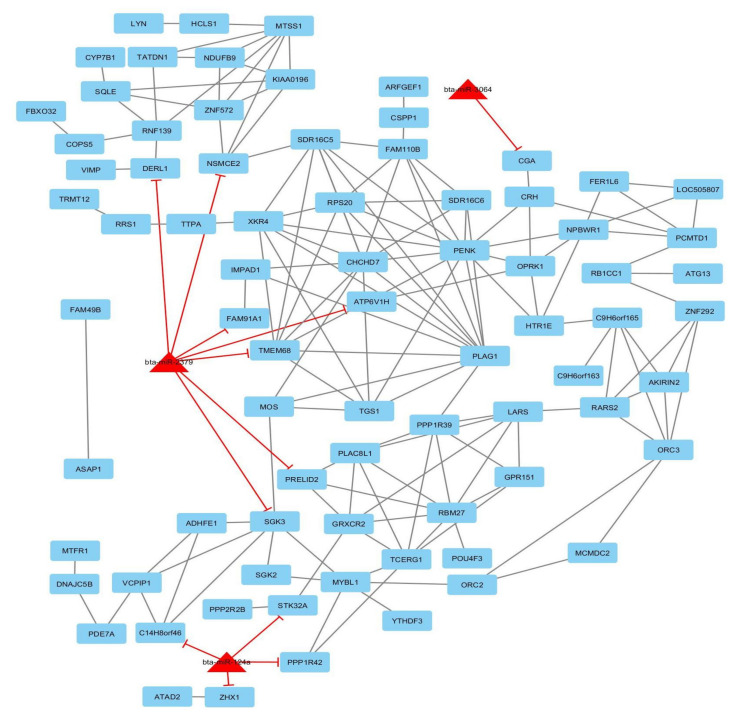
The network of carcass weight related genes and miRNAs using Cytoscape software (version 3.7.2; www.cytoscape.org); each node denotes a gene (rectangle) and miRNA (triangle) and edges depict miRNA–gene relationships.

The global gene-traits network of all related genes and traits was rebuilt. The reconstructed network includes 366 genes and five traits as nodes and 486 edges between traits and related genes (Figure S1). Twenty-three genes were common between the three traits (CW, EMA, and YW), 61 genes were common between CW and YW, 2 genes were common between BT and EMA, 1 gene was common between BT and MS and the other genes were not common between traits. For the bipartite network (miRNA-gene), we compiled candidate genes and miRNAs (nodes) involved in each trait based on literature mining and PPI resources that were abstracted: BIND, DIP, BioGRID, MIPS, STRING, GRNs, and miRNA–targeted genes. Briefly, miRNAs–gene networks are commonly represented in an undirected graph format, with nodes representing miRNAs or genes and edges representing interactions (genes–genes and miRNAs–targeted genes). Based on the current knowledge of interactions in databases, the interactions were detected for nodes and edges in the candidate genes and miRNAs for each trait separately. Five major miRNA–gene networks are illustrated in Figure 3, Figure 4 and Figures S2–S4 (for BT, 50 genes and 2 miRNAs as nodes and 75 interactions as edges; for CW, 78 genes and 3 miRNAs as nodes and 163 interactions as edges; for EMA, 31 genes and 1 miRNAs as nodes and 58 interactions as edges; for MS, 15 genes and 1 miRNAs as nodes and 16 interactions as edges; for YW, 78 genes and 3 miRNAs as nodes and 144 interactions as edges were observed).

## 4. Discussion

Carcass traits and growth traits, such as yearling weight, have a crucial role in livestock due to their influence on meat production. Therefore, it is important to identify the genomic regions that contribute most to the genetic variations for these traits. This study is the first to report a weighted single-step genome-wide association study of five economically important traits (backfat thickness, carcass weight, eye muscle area, marbling score, and yearling weight) in Hanwoo cattle. The weighted single-step method is useful for GWAS because it allows one to integrate the information of genotyped and non-genotyped animals simultaneously, and the use of different weights for SNPs according to their importance for the trait of interest, which leads to improving QTL detection [10]. In addition, this method provides the possibility to work with sets of consecutive SNPs (SNP windows), which can be effective in finding QTL regions due to linkage disequilibrium (LD) compared to single-SNP analysis [12]. In fact, when the number of animals with both phenotypes and genotypes is relatively small, the WssGWAS is a relatively better method than classical GWAS for improving the statistical power of QTL detection. Furthermore, the combination of GWAS results and pathway and gene network analyses provide additional insights into the ability to identify genomic regions influencing these traits, especially when the detected genes are involved in various biological processes. In other words, systems biology is a systemic level approach to study an all-around understanding of complicated biological systems outside the molecular-level scale [52]. Instead of analyzing individual components or aspects of the organism, such as a cell nucleus or metabolism, systems biologists focus on all aspects and the interactions between them as part of one system. These roles are eventually responsible for an organism’s form and its functions. Unlike previous GWAS studies, the present study identifies genes and miRNAs networks within different traits. This study proposes a computational method to detect the integration of weighted single-step GWAS and bipartite networks; that is, groups of detected miRNAs and their detected target genes, that are believed to take part in post-transcriptional gene regulation in different traits, such as BT, CW, EMA, MS, and YW. Therefore, this method of reconstructing miRNA–gene networks could help to clarify the complicated biological procedures.

In the WssGWAS analysis, thirty-three QTL regions (window of 20 adjacent SNPs) were associated with at least one of the five traits. These regions are distributed on chromosomes 1, 2, 5, 6, 7, 9, 10, 11, 13, 14, 16, 19, 23, and 27 (Table 2). The largest number of significant genomic regions associated with different traits was found on chromosome 14 (10 QTLs), followed by chromosome 6 (6 QTLs), explaining a relatively large percentage of the total additive genetic variation. The results obtained from the WssGWAS for BT (Table 2) indicated that the trait-specific windows with large-effect are located on chromosomes 2, 7, 11, 13 and 16. One-hundred genes were annotated in these genomic regions according to NCBI, some of which have been previously reported to be involved in metabolism and transport of lipids, and comprise the PRELID1, FGFR4, COMMD1, PFN3 and RGS14 genes (Table 3). For BT, the large-effect window was located in the region of 39.69–40.83 Mb on chromosome 7, explaining 2.59% of additive genetic variance where the five promising candidate genes and their biological functions were highlighted. For instance, the fibroblast growth factor receptor 4 (FGFR4) gene is a transmembrane tyrosine kinase receptor that may play essential roles in the regulation of hepatic bile acid and lipid metabolism [53]. PRELID1 (PRELI Domain Containing 1), another gene located in the same region, is a protein-coding gene that regulates lipid accumulation in the mitochondria by shuttling phospholipids in a lipid-specific manner across the intermembrane space [54]. Other important genes within this window include HK3 (Hexokinase 3), which is crucial for glucose metabolism pathways and phosphorylates glucose to produce glucose-6-phosphate; the RGS14 (Regulator of G Protein Signaling 14) gene, which encodes a member of the regulator of G-protein signaling family and as a GTPase activating protein (GAP), it increases the rate of conversion of GTP to GDP; PDLIM7 (PDZ and LIM domain 7), which is known to regulate muscle development and function, and associates with—and localizes to—actin filaments in fibroblasts via its PDZ domain [55]. A positional candidate gene located on chromosome 2 in the position of 72.46–73.83 Mb is INHBB (Inhibin Subunit Beta B), which displayed high expression in human adipocytes [56] and thus is probably related to BT trait. Our results differ from those reported by Mokry et al. [57] who detected significant SNPs for BT on chromosomes 1, 3, 10, 13, and 19 of Canchim animals. Similarly, Lee et al. [58] described regions containing potential QTLs for BT on chromosomes 13 and 16 of Hanwoo cattle; however, the regions were distant from those found on chromosomes 13 and 16 in the present study. Moreover, other regions on chromosomes 6, 10, 13, 14, 17, and 22 using the three GWAS approaches have previously been reported to be likely associated with BT in a composite beef cattle breed [59].

In the BT network, bta-miR-124a and bta-miR-2379 suppressed the EXO1 gene that encodes a protein with RNase H activity and 3’ to 5’ exonuclease activity. In addition, bta-miR-124a suppressed the CLASP1 and NIFK genes, and bta-miR-2379 suppressed the NIPA1, PRELID1, RALGAPA2, and SLC31A1 genes. The NIFK gene is a hub gene that encodes a protein that interacts with the other proteins, such as a protein that may bind RNA and play a role in the cell-cycle process. Gene Ontology, KEGG and Reactome pathway analyses for this trait include lipid transport (the directed motion of lipids into, within, out of, or between cells or a cell); lipid localization (the maintenance or transportation of lipids to a specific location); lipid transporter activity; lipid metabolic process (the pathways involving lipids and chemical reactions which includes fatty acids, soaps, waxes, and other long-chain bases); lipid binding (interacting non-covalently and selectively with a lipid); lipid biosynthetic process (the pathways and chemical reactions leading to the formation of lipids); the Rap1 signaling pathway (the pathway that controls diverse procedures, such as cell–cell junction formation, cell adhesion, and cell polarity, which also regulates MAPK (MAP kinase) activity).

Concerning CW, eleven large-effect windows (explaining more than 1% of additive genetic variance) were identified, in order of significance, on chromosomes 14, 9, and 7. Of these, seven genomic regions on chromosome 14 are probably pleiotropic QTLs, which are located at positions 16 Mb, 22 Mb, 24 Mb, 25 Mb, 29 Mb, 30 Mb, and 32 Mb. Only pleiotropic QTLs located in the positions of 22.09–23.61 Mb and 24.58–25.33 Mb were common between CW, EMA, and YW; the other pleiotropic QTLs were to influence CW and YW. A total of 136 genes were annotated in these genomic regions for CW and, according to NCBI, some of them were associated with EMA and YW. This was expected due to the high positive genetic correlation between CW and YW (0.76) and between CW and EMA (0.56), which was estimated in Hanwoo cattle [31], indicating that the same genes were controlling these traits. The most relevant pleiotropic QTL was identified on chromosome 14 at 24.58–25.33 Mb, which explained 17.66% of additive genetic variance in CW, 7.98% in EMA, and 9.96% in YW. These were harbored by 12 candidate genes (Table 2), among which XKR4, TMEM68, LYN, PLAG1, CHCHD7, and PENK were noteworthy. This QTL window was also detected in several association studies on carcass and growth traits in beef cattle, which confirms the reliability of our results. For instance, previous studies reported genetic variants in PLAG1 (pleiomorphic adenoma gene 1) for their associations with bovine stature [60], carcass weight [4,28], birth weight [61], in addition to early life bodyweight and peripubertal weight [62] in different cattle breeds. The PLAG1 gene has been associated with meat tenderness in Nellore cattle [13] and has a pleiotropic effect and can, therefore, be an excellent candidate for a large-effect gene because it acts on different traits.

In an earlier study on Hanwoo cattle using the Illumina BovineSNP50 BeadChip, Lee et al. [28] detected potential candidate genes, such as PLAG1, CHCHD7, and FAM110B, that were associated with CW, confirming our findings. Other genes, located on chromosome 14 at 24 Mb, contains LYN, XKR4, and TMEM68, which have been associated with feed intake, growth and meat tenderness in cattle [13,63]. The XKR4 gene has been reported to be associated with rump fat thickness in indicine and composite cattle [64]. Another study also identified that TGS1 and LYN genes influenced carcass traits in cattle [65] and the PENK gene has been associated with fat thickness and intramuscular fat in a composite beef cattle breed [59]. Further important genes, located on other regions of chromosome 14 include the ASAP1 gene, related to meat quality traits in three French beef cattle breeds [66]; the SGK3 gene, which is involved in the regulation of cell growth and growth according to gene ontology (Table 3); the CRH gene that plays important roles for growth and development in mammals, including cattle. Bhuiyan et al. [25] stated that two coding SNPs (synonymous and missense) in CRH are significantly related to the CW in Hanwoo, and another study of the same breed [67] reported that a missense mutation of CRH was significantly associated with the EMA. According to our findings and previous studies, most discovered QTLs related to many economically important traits in cattle have been observed on chromosome 14. Several genome-wide significant QTL on chromosome 14 for growth, milk, and meat production traits in cattle have been published in recent years. For instance, pleiotropic QTL located at 23–26 Mb was associated with body weight and calving ease in Brangus, Gelbvieh, and Simmental cattle [68]. For backfat thickness and rump fat thickness, QTL have been reported on chromosome 14 in Bos indicus and Bos taurus cattle by Bolormaa et al. [69]. The region on chromosome 14 showing an association with milk fatty acid is the region harboring DGAT1, which is known to influence milk production traits [70] and milk fat composition [71]. Hence, chromosome 14 is a hot spot for several causal variants among chromosomes for the studied trait.

A positional candidate gene on chromosome 7 (58.66–60.49 Mb) is SH3RF2 (also known as PPP1R39), which, like the myostatin gene, negatively regulates muscular tissue growth in the event of low expression levels related to muscular hypertrophy. In a study in the Blonde d’Aquitane breed, the authors revealed that the SH3RF2 gene was identified as one of the genes responsible for the double muscle presence in animals [72]. Some of the identified candidate genes in genomic regions act as olfactory receptors, including the genes located in the window of chromosome 2 at the 0.35–1.28 Mb position (LOC104971094, LOC107132231, LOC107132232, and LOC784948) in the BT trait, in addition to LOC100138092 and LOC788619 located in the window of chromosome 7 at 58.66–60.49 Mb for the CW trait. Olfactory receptors contribute to the change of GDP (guanosine diphosphate) to GTP (guanosine triphosphate), which are regulators of G proteins, since GDP and GTP can be used as energy sources and are responsible for transferring energy within the cell [73]. Moreover, olfactory receptors are known to act on adipose tissue and adipocyte differentiation, increasing fat accumulation [74], consequently affecting meat traits. One region for CW was identified in the region of 62.93–64.58 Mb on chromosome 9 and harbors the positional candidate gene AKIRIN2. This gene is a nuclear factor and previous reports showed that the AKIRIN2 was a potential functional candidate gene for meat quality in pigs and beef cattle [75,76,77].

Regarding the CW network, bta-miR-124a suppressed C14H8orf46, PPP1R42, STK32A, and ZHX1 genes; bta-miR-2379 suppressed ATP6V1H, DERL1, FAM91A1, NSMCE2, PRELID2, SGK3, and TMEM68 genes; bta-miR-3064 suppressed the CGA gene. The PLAG1 (Pleomorphic adenoma gene 1) gene is a hub gene that encodes a zinc finger protein (ZFP) with nuclear localization signals [78]. Gene Ontology, KEGG, and Reactome pathways analysis for this trait included the regulation of cell growth (any process that modulates the rate, extent, frequency, and direction of cell growth); epidermal growth factor receptor signaling pathway (sets of molecular signals initiated by binding of a ligand to the tyrosine kinase EGFR receptor (ERBB1) on the surface of a cell whose pathway ends with the regulation of a downstream cellular procedure, such as transcription [79]); cell growth (the biological process whereby a cell increases in size over time through the biosynthetic and accretion production of matter similar to that already present); multicellular organism growth (the enhancement in size of a whole multicellular organism, in contrast to cell growth); organ growth (the enhancement in mass or size of an organ); growth; developmental growth (the enhancement in mass or size of whole organism, a part of a cell or an organism that has the specific outcome of the development of the organism over time) [80]; protein binding (interacting non-covalently and selectively with other protein complexes or protein); neuroactive ligand–receptor interaction [81] (all known genes encoding receptors for each ligand were detected from this ligand–receptor interaction list).

Analyses for EMA identified seven relevant QTL regions on chromosomes 1, 6, 9, 14, and 19, of which seventy-two genes were annotated in these genomic regions. A positional candidate gene for EMA on chromosome 19 (48.90–50.02 Mb) is PITPNC1, which acts as a cytoplasmic phosphatidylinositol transfer protein and is involved in lipid transport [82], and the ZBTB38 gene (Zinc finger and BTB domain containing 38) located in the region of 127.77–128.74 Mb on chromosome 1 is an important candidate gene for the selection of body measurement traits in native Chinese cattle breeds through marker-assisted selection [83].

In the EMA network, bta-miR-2379 likely suppressed ATP6V1H, SMURF2, and TMEM68 genes. The PLAG1 (Pleomorphic adenoma gene 1) gene is a hub gene in this network similar to the CW network. Gene Ontology, KEGG, and Reactome pathway analyses for this trait included lipid metabolic process (the pathways and chemical reactions related to lipids); regulation of the lipid metabolic process (the biological process which modulates the extent of the pathways and chemical reactions involving lipids); response to lipids (the biological process which results in an alteration in activity or state of an organism or a cell); cell adhesion molecules (they are proteins or glycoproteins expressed on the cell surface that play an important role in a large array of biological processes, including inflammation, hemostasis, embryogenesis, the immune response, and the development of neuronal tissue).

The thirteen windows with large effects obtained for YW are located on chromosomes 2, 6, 10, and 14, and according to NCBI, 128 genes were annotated in these regions, 88 of which were common to CW as mentioned in Table 2. Moreover, there are numerous candidate genes on chromosome 6 in the region of 37.26–38.45 Mb associated with growth traits in cattle, among which PPM1K, ABCG2, PKD2, SPP1, MEPE, and IBSP were notable in this study. PPM1K has been associated with increased mid-point metabolic weight and carcass weight and also decreased residual feed intake, feed efficiency conversion ratio, and marbling score [84]. This gene plays a key role in cellular survival and development by regulating mitochondrial permeability transition pore function, as well as a complicated phosphorus metabolic process [85]. The ABCG2 gene (ATP-binding cassette, subfamily G, member 2) is known to be involved in fat synthesis and showed associations with weaning weight, yearling weight, and direct birth weight in Brangus beef cattle [6], as well as milk composition in dairy cattle [86]. The PKD2 gene (polycystic kidney disease 2) affects body weight in Australian Merino sheep [87]. Other genes containing the SPP1 gene (secreted phosphoprotein 1) that have been reported to be associated with carcass weight, yearling weight, and post-weaning growth [88] include the IBSP gene (integrin-binding sialo protein), which is involved in the processing of the bone mineralization; MEPE gene (Matrix extracellular phosphoglyco protein), which demonstrates different expressions in the RNA-Seq analysis of heifer pregnancy from pre- and post-puberty Brangus heifers [89]. Another significant QTL region for YW was identified on chromosome 6 at 44.67–45.42 Mb, which harbors the PPARGC1A gene and is considered to be a positional candidate gene for carcass traits in Hanwoo cattle [25,90]. PPARGC1A (Peroxisome proliferator-activated receptor-γ coactivator-1α) plays a significant role in many aspects of glucose and fat metabolism and energy balance. It has also been demonstrated that PPARGC1A was significantly associated with body weight and average daily gain in Chinese native cattle [91], milk fat yield in Holstein cattle [92], as well as with growth, and meat quality traits in Brangus steers [93].

Consistent with our findings, some previous studies have identified genes in relevant QTL regions on chromosome 6 that are associated with various traits in different cattle breeds. For example, Snelling et al. [94] found that most SNPs associated with direct growth are located on chromosome 6 in crossbred beef cattle. In another study across ten US cattle breeds using 50K SNP chip data, Saatchi et al. [68] also identified a large-effect pleiotropic QTL located on this chromosome at 37–42 Mb. Besides, other studies have reported the QTL region on chromosome 6 related to average daily gain in Nellore cattle [95], milk production [96] and reproductive traits [97] in dairy cattle.

For the YW network, it is possible that bta-miR-124a suppressed C14H8orf46, PPARGC1A, and PPP1R42 genes, bta-miR-2379 suppressed ATP6V1H, FAM91A1, NSMCE2, SGK3, and TMEM68 genes and bta-miR-3064 suppressed RNF111 and SLTM genes. PENK gene is a hub gene that encodes a pre-proprotein, which is proteolytically processed to produce multiple protein products. Gene Ontology, KEGG, and Reactome pathways analyses for this trait included regulation of cell growth; cell growth; multicellular organism growth; organ growth; growth; developmental growth; response to growth factor (the biological process which results in an alteration in activity or state of an organism or a cell); cellular response to growth factor stimulus; protein binding; catalytic activity on a protein (catalytic activity that acts to change and modify a protein).

The GWAS of MS using the weighted single-step method resulted in five significant QTL windows located on chromosome 5, 14, 23, and 27, which were trait-specific QTL for the MS trait (Table 2). These regions explained a relatively high percentage of genetic variance for MS and fifty genes were also annotated according to NCBI. The largest QTL window, which explained 2.25% of genetic variance, was captured on chromosome 5 (95.87–97.74 Mb) and harbors a number of candidate genes (APOLD1 and CREBL2) involved in the metabolism and transport of lipids (Table 3). Magalhães et al. [13] identified two large-effect windows associated with MS on chromosome 5 of Nellore Cattle, but the regions were distant from those observed in the present study. Additionally, potential QTL regions associated with marbling score in Hanwoo cattle have been recently identified on chromosomes 2, 12, 16, and 24 by Bedhane et al. [24], which differ with the regions identified in our study. Among the identified genes for MS on chromosome 14 at the position 5.01–5.69 Mb, COL22A1 has been previously reported to be associated with milk fat percentage and milk yield in the Chinese Holstein population [98]. A positional candidate gene located in the region of 19.17–20.48 Mb on chromosome 27 is FGF20 (fibroblast growth factor 20), which is a member of the FGF family, and belongs to the FGF9 subfamily with abundant roles in development, organogenesis, tissue repair, homeostasis, and tissue fibrosis [99].

Additionally, in the MS network, bta-miR-124a suppressed ABT1 genes. Bta-miR-2379 suppressed ATP6V1H, FAM91A1, NSMCE2, SGK3, and TMEM68 genes, and bta-miR-3064 suppressed RNF111 and SLTM genes. HIST1H2AG gene is a hub gene consisting of basic nuclear proteins and is responsible for the nucleosome structure of the chromosomal fiber in eukaryotes. Gene Ontology, KEGG, and Reactome pathways analyses for this trait included lipid metabolic process; lipid transport; lipid biosynthetic process; lipid storage (the maintenance and accumulation in lipid tissues or cells. Lipid source can be accumulated during the early developmental phase for utilization and mobilization in a later phase of development, fat cell differentiation (the biological process in that an unspecialized cell saves specialized features of an adipocyte, an animal connective tissue cell specialized for the storage and synthesis of fat), protein–lipid complex binding (interacting non-covalently and selectively with a protein–lipid complex), and in the MAPK signaling pathway (the MAPK (mitogen-activated protein kinase) cascade is a highly conserved module which is involved in various cellular functions, including cell differentiation, cell proliferation, and cell migration).

## 5. Conclusions

To our knowledge, this is the first report on the integration of weighted single-step GWAS and network analyses for yearling weight and carcass traits in Hanwoo cattle, which revealed thirty-three relevant genomic regions related to the traits of interest. Significant enrichments for biological processes and KEGG pathway analyses, including the lipid biosynthetic process, regulation of the lipid metabolic process, transport or localization of lipids, regulation of growth, developmental growth, and multicellular organism growth were obtained. It is interesting to note that a number of the common genes we detected are involved in metabolic and cellular processes that have possible impacts on the studied traits. These results suggest that the incorporation of GWAS results and bipartite network analyses can illuminate the genetic architecture underlying complex traits. This method could enhance the identification of biological mechanisms and respective candidate genes, and in addition, could be useful in breeding schemes aimed at improving growth and carcass traits in Hanwoo beef cattle.

## Figures and Tables

**Table 1:** Summary statistics for phenotypic data used to estimate variance components in Hanwoo cattle.

Trait (Units)	Number of Animals with Record (and Genotype)	Mean (SE)	Min.	Max.	SD	CV%
BT (mm)	5824 (1151)	8.71 (0.05)	1.00	30.00	3.71	42.61
CW (kg)	5824 (1151)	343.96 (0.60)	158.00	519.00	45.61	13.26
EMA (cm^2^)	5821 (1151)	78.90 (0.12)	40.00	123.00	9.12	11.56
MS (score)	3991 (1151)	3.33 (0.03)	1.00	9.00	1.61	48.46
YW (kg)	15,279 (1540)	342.06 (0.38)	133.86	535.90	47.48	13.88

BT, Backfat Thickness; CW, Carcass Weight; EMA, Eye Muscle Area; MS, Marbling Score; YW, Yearling Weight.

**Table 2:** Summary of SNP windows that explained >1% of genetic variance for all studied traits in Hanwoo cattle, with a list of annotated genes within each window.

Trait ^a^	Chr ^b^	QTL Region (Mb) ^c^	GV% ^d^	Candidate Genes ^e^
BT	2	0.35–1.28	2.16	LOC104971094, LOC107132231, LOC107132232, LOC784948, LGSN, OCA2, LOC783772, LOC100301143, HERC2, LOC104971093, TRNAE-CUC, NIPA1, NIPA2, LOC107132230, CYFIP1, TUBGCP5, TRNAE-UUC, CCDC115, IMP4, PTPN18
BT	2	72.46–73.83	1.11	INHBB, LOC781979, LOC104971258, GLI2, LOC104971259, LOC100335292, TRNAL-CAA, TFCP2L1, CLASP1, NIFK, TSN
BT	7	39.69–40.83	2.59	HK3, UIMC1, LOC510252, LOC100141185, LOC101905975, LOC784341, LOC533921, LOC782447, TRNAR-CCU, ZNF346, FGFR4, LOC100336707, LOC540197, RAB24, PRELID1, MXD3, LMAN2, RGS14, SLC34A1, PFN3, F12, GRK6, PRR7, DBN1, PDLIM7, DOK3, LOC104969157, DDX41, FAM193B, LOC509184, LOC100139419, TMED9, B4GALT7, LOC107132625, N4BP3, RMND5B, LOC101905866, NHP2, HNRNPAB, PHYKPL, COL23A1
BT	11	60.34–61.55	1.69	FAM161A, CCT4, COMMD1, B3GNT2, TMEM17, EHBP1, OTX1
BT	13	40.02–41.40	1.08	CFAP61, INSM1, RALGAPA2, LOC104973781, LOC104973782, KIZ, LOC100140493, XRN2, NKX2-4, NKX2-2, LOC614124, PAX1
BT	16	34.80–35.91	1.08	LOC100297170, PLD5, LOC104974409, LOC104974410, BECN2, MAP1LC3C, EXO1, WDR64, LOC101907876
CW	7	58.66–60.49	1.91	PRELID2, LOC107132647, LOC100138092, LOC788619, GRXCR2, SH3RF2, PLAC8L1, LARS, RBM27, POU4F3, TCERG1, GPR151, PPP2R2B, TRNAC-GCA, STK32A
CW	9	62.93–64.58	1.98	TRNAE-UUC, LOC100336843, AKIRIN2, ORC3, RARS2, SLC35A1, CFAP206, C9H6orf163, SMIM8, LOC104969583, GJB7, LOC107132774, ZNF292, LOC509829, CGA, HTR1E
CW	14	11.23–12.26	1.04	ASAP1, FAM49B, GSDMC
CW	14	16.55–17.80	4.46	NSMCE2, KIAA0196, SQLE, ZNF572, MTSS1, NDUFB9, TATDN1, LOC104968469, RNF139, TRMT12, LOC531462, TMEM65, FER1L6, LOC101907615, FAM91A1, ANXA13
CW	14	17.85–19.46	2.00	LOC100848930, FBXO32, WDYHV1, ATAD2, ZHX1, C14H8orf76, FAM83A, TRNAM-CAU, LOC104974006, TBC1D31, DERL1, ZHX2, LOC104974007, LOC100139328
CW	14	22.09–23.61	4.31	SNTG1, LOC614437, PCMTD1, LOC101906226, LOC104974020, ST18, LOC100141260, LOC101906592, FAM150A, RB1CC1, LOC104974017, NPBWR1, OPRK1, ATP6V1H, RGS20
CW	14	24.58–25.33	17.66	XKR4, TMEM68, TGS1, LYN, RPS20, MOS, PLAG1, CHCHD7, SDR16C5, SDR16C6, PENK, LOC101907667
CW	14	25.36–26.15	2.43	LOC101907667, IMPAD1, FAM110B
CW	14	29.43–30.44	3.75	NKAIN3, LOC107133118, GGH, TTPA, YTHDF3, LOC101907975
CW	14	30.54–32.16	1.30	MIR124A-2, BHLHE22, CYP7B1, LOC104974032, ARMC1, MTFR1, LOC104974034, PDE7A, LOC101902754, LOC100299601, LOC104974036, DNAJC5B, TRNAY-GUA, TRNAA-AGC, TRIM55
CW	14	32.25–33.90	1.43	CRH, LOC790324, ZSCAN5B, RRS1, ADHFE1, C14H8orf46, MYBL1, VCPIP1, SGK3, LOC104974037, MCMDC2, LOC784087, LOC100847363, TCF24, PPP1R42, COPS5, CSPP1, ARFGEF1, TRNAC-GCA, CPA6, LOC101902584
EMA	1	127.77–128.74	2.27	GK5, TFDP2, LOC101903974, LOC511302, ATP1B3, GRK7, RNF7, LOC104968752, RASA2, LOC100294923, ZBTB38, LOC107131348, LOC104971030, LOC104971031, PXYLP1, LOC104971032
EMA	6	0.1–1.03	1.00	APELA, LOC101905490, LOC513842, LOC101907917
EMA	6	54.52–55.64	1.11	NOT_FOUND
EMA	9	57.18–58.10	2.22	TRNAC-ACA, LOC782527, EPHA7
EMA	14	22.09–23.61	1.75	SNTG1, LOC614437, PCMTD1, LOC101906226, LOC104974020, ST18, LOC100141260, LOC101906592, FAM150A, RB1CC1, LOC104974017, NPBWR1, OPRK1, ATP6V1H, RGS20
EMA	14	24.58–25.33	7.98	XKR4, TMEM68, TGS1, LYN, RPS20, MOS, PLAG1, CHCHD7, SDR16C5, SDR16C6, PENK, LOC101907667
EMA	19	48.90–50.02	1.00	LOC100140873, TEX2, TRNAG-UCC, LOC104975109, LOC101902037, PECAM1, MILR1, POLG2, DDX5, MIR3064, CEP95, SMURF2, TRNAE-CUC, KPNA2, TRNAR-CCG, C19H17orf58, BPTF, TRNAE-UUC, NOL11, TRNAS-AGA, PITPNC1, LOC101905668
MS	5	95.87–97.74	2.24	ATF7IP, LOC100139060, GRIN2B, EMP1, GSG1, FAM234B, HEBP1, GPRC5D, GPRC5A, DDX47, APOLD1, CDKN1B, LOC101901926, GPR19, CREBL2, LOC101902028, LOC107132517, DUSP16
MS	14	5.01–5.69	1.00	LOC100850800, COL22A1, FAM135B
MS	23	30.27–31.28	2.03	ZNF389, ZSCAN16, ZNF165, OR2B6, HIST1H2BB, HIST1H2AG, bta-mir-2379, ZNF184, ZNF391, POM121L2, PRSS16, HIST1H2BN, ZNF322, ABT1
MS	27	16.11–17.15	1.28	LOC101905556, LOC101905700, ZFP42, TRNAG-UCC, TRIML2, TRNAF-AAA, TRIML1, LOC507011
MS	27	19.17–20.48	1.46	MICU3, FGF20, LOC104976064, TRNAC-ACA, MSR1, LOC104976066, TUSC3
YW	2	42.77–43.75	1.71	LOC785568, LOC615401, ARL6IP6, TRNAY-GUA, PRPF40A, FMNL2, LOC101902790
YW	6	37.26–38.45	1.36	FAM13A, LOC104972724, LOC100847719, HERC3, NAP1L5, PYURF, PIGY, HERC5, HERC6, PPM1K, ABCG2, LOC781421, PKD2, SPP1, MEPE, IBSP, LOC104972726
YW	6	39.50–40.67	2.68	LOC782905
YW	6	44.67–45.42	1.14	PPARGC1A
YW	6	48.80–49.97	2.30	LOC107132565
YW	10	50.75–51.96	1.56	LOC107132854, FAM81A, MYO1E, CCNB2, RNF111, SLTM, FAM63B, LOC533308, ADAM10, LIPC, LOC101904602, LOC101903685, TRNAE-UUC
YW	14	16.55–17.80	1.70	NSMCE2, KIAA0196, SQLE, ZNF572, MTSS1, NDUFB9, TATDN1, LOC104968469, RNF139, TRMT12, LOC531462, TMEM65, FER1L6, LOC101907615, FAM91A1, ANXA13
YW	14	22.09–23.61	2.54	SNTG1, LOC614437, PCMTD1, LOC101906226, LOC104974020, ST18, LOC100141260, LOC101906592, FAM150A, RB1CC1, LOC104974017, NPBWR1, OPRK1, ATP6V1H, RGS20
YW	14	24.58–25.33	9.96	XKR4, TMEM68, TGS1, LYN, RPS20, MOS, PLAG1, CHCHD7, SDR16C5, SDR16C6, PENK, LOC101907667
YW	14	25.36–26.15	1.74	LOC101907667, IMPAD1, FAM110B
YW	14	29.43–30.44	2.64	NKAIN3, LOC107133118, GGH, TTPA, YTHDF3, LOC101907975
YW	14	30.54–32.16	2.62	MIR124A-2, BHLHE22, CYP7B1, LOC104974032, ARMC1, MTFR1, LOC104974034, PDE7A, LOC101902754, LOC100299601, LOC104974036, DNAJC5B, TRNAY-GUA, TRNAA-AGC, TRIM55
YW	14	32.25–33.90	1.43	CRH, LOC790324, ZSCAN5B, RRS1, ADHFE1, C14H8orf46, MYBL1, VCPIP1, SGK3, LOC104974037, MCMDC2, LOC784087, LOC100847363, TCF24, PPP1R42, COPS5, CSPP1, ARFGEF1, TRNAC-GCA, CPA6, LOC101902584

^a^ BT, Backfat Thickness; CW, Carcass Weight; EMA, Eye Muscle Area; MS, Marbling Score; YW, Yearling Weight. ^b^ Chromosome. ^c^ Position of QTL region. ^d^ Percentage of genetic variance explained by 20 SNPs windows. ^e^ Genes identified according to genome assembly UMD_3.1.

**Table 3:** Gene Ontology, KEGG and Reactome pathways where the candidate genes are significantly enriched (*p* < 0.05).

Traits ^a^	Term ID	Term Name	Genes	*p*-Value
BT	GO:0006869	lipid transport	PRELID1	0.010233961
	GO:0010876	lipid localization	PRELID1	0.014172362
	GO:0005319	lipid transporter activity	PRELID1	0.019846208
	GO:0006629	lipid metabolic process	FGFR4	0.025006995
	GO:0008289	lipid binding	COMMD1, PFN3	0.047311441
	GO:0008610	lipid biosynthetic process	FGFR4	0.035303187
	KEGG:04015	Rap1 signaling pathway	RGS14	0.047961872
CW	GO:0001558	regulation of cell growth	SGK3	0.044446017
	GO:0007173	epidermal growth factor receptor signaling pathway	FAM83A	0.039752933
	GO:0016049	cell growth	POU4F3	0.035059849
	GO:0035264	multicellular organism growth	PLAG1	0.030366765
	GO:0035265	organ growth	PLAG1, CGA	0.025673681
	GO:0040007	growth	PLAG1, POU4F3, CGA, SGK3	0.020980597
	GO:0048589	developmental growth	PLAG1, POU4F3, CGA	0.016287514
	GO:0060560	developmental growth involved in morphogenesis	POU4F3	0.01159443
	GO:0005515	protein binding	ARFGEF1, ATAD2, LYN, MYBL1, RB1CC1, AKIRIN2, CRH, CGA, NPBWR1, PENK, PPP1R42, RGS20, TCERG1, ZHX1, ZHX2	0.006901346
	KEGG:04080	Neuroactive ligand-receptor interaction	CGA, HTR1E, NPBWR1, OPRK1, PENK, CRH	0.039862933
EMA	GO:0006629	lipid metabolic process	LYN, SDR16C5	0.049805624
	GO:0019216	regulation of lipid metabolic process	LYN	0.042803519
	GO:0033993	response to lipid	DDX5	0.043747312
	GO:0008289	lipid binding	TEX2	0.049508111
	KEGG:04514	Cell adhesion molecules	LOC100140873, PECAM1	0.04067771
	REAC:R-BTA-210990	PECAM1 interactions	LYN, PECAM1	0.049805624
MS	GO:0006629	lipid metabolic process	CREBL2	0.01889403
	GO:0006869	lipid transport	APOLD1, MSR1	0.041826385
	GO:0008610	lipid biosynthetic process	CREBL2	0.021157549
	GO:0010876	lipid localization	APOLD1	0.016791777
	GO:0019915	lipid storage	MSR1	0.025742261
	GO:0045444	fat cell differentiation	CREBL2	0.019486141
	GO:0008289	lipid transport	APOLD1	0.017945142
	GO:0071814	protein-lipid complex binding	MSR1	0.016404143
	KEGG:04010	MAPK signaling pathway	DUSP16, FGF20	0.014863145
	REAC:R-BTA-5673001	RAF/MAP kinase cascade	DUSP16, FGF20, GRIN2B	0.016791777
YW	GO:0001558	regulation of cell growth	ADAM10, SGK3	0.046130564
	GO:0016049	cell growth	ADAM10	0.043928692
	GO:0035264	multicellular organism growth	PLAG1	0.04172682
	GO:0035265	organ growth	PLAG1	0.039524949
	GO:0040007	growth	ADAM10, PLAG1, SGK3	0.037323077
	GO:0040008	regulation of growth	ADAM10, SGK3, MYO1E	0.035121205
	GO:0048589	developmental growth	PLAG1	0.032919333
	GO:0070848	response to growth factor	IBSP, RNF111	0.030717462
	GO:0071363	cellular response to growth factor stimulus	RNF111	0.02851559
	GO:0005515	protein binding	ADAM10, ARFGEF1, LYN, MYBL1, PPARGC1A, RB1CC1, CRH, MEPE, NPBWR1, PKD2, PENK, PPP1R24, RGS20, SPP1	0.026313718
	GO:0140096	catalytic activity, acting on a protein	ADAM10, CCNB2, COPS5, CPA6, GGH, HERC3, HERC5, HERC6, LYN, MOS, PCMTD1, PPM1K, RNF111, RNF139, SGK3, VCPIP1, NSMCE2	0.048130564

^a^ BT, Backfat Thickness; CW, Carcass Weight; EMA, Eye Muscle Area; MS, Marbling Score; YW, Yearling Weight.

## Supplementary Materials

The following are available online at https://www.mdpi.com/2076-2615/10/10/1836/s1: Figure S1: The global network of all related genes and traits using Cytoscape software (version 3.7.2; www.cytoscape.org). Oval nodes indicate genes and rectangle nodes indicate traits, Figure S2: The network of eye muscle area related genes and miRNA using Cytoscape software (version 3.7.2; www.cytoscape.org); each node denotes a gene (rectangle) and miRNA (triangle) and edges depict miRNA-gene relationships, Figure S3: The network of marbling score related genes and miRNAs using Cytoscape software (version 3.7.2; www.cytoscape.org); each node denotes a gene (rectangle) and miRNA (triangle) and edges depict miRNA–gene relationships, Figure S4: The network of yearling weight related genes and miRNAs using Cytoscape software (version 3.7.2; www.cytoscape.org); each node denotes a gene (rectangle) and miRNA (triangle) and edges depict miRNA–gene relationships.
