# Supplementary material for: Integrating Single-Step GWAS and Bipartite Networks Reconstruction Provides Novel Insights into Yearling Weight and Carcass Traits in Hanwoo Beef Cattle
Source: Animals (Basel). 2020 Oct 9;10(10):1836. doi: 10.3390/ani10101836 (PMC7601430; doi:10.3390/ani10101836)
Supplement: Supplementary file 1 [file animals-10-01836-s001.pdf]

animals

MDPI

Article

Integrating single-step GWAS and bipartite networks reconstruction provides novel insights into yearling weight and carcass traits in Hanwoo beef cattle

Masoumeh Naserkheil, Abolfazl Bahrami, Deukhwan Lee and Hossein Mehrban

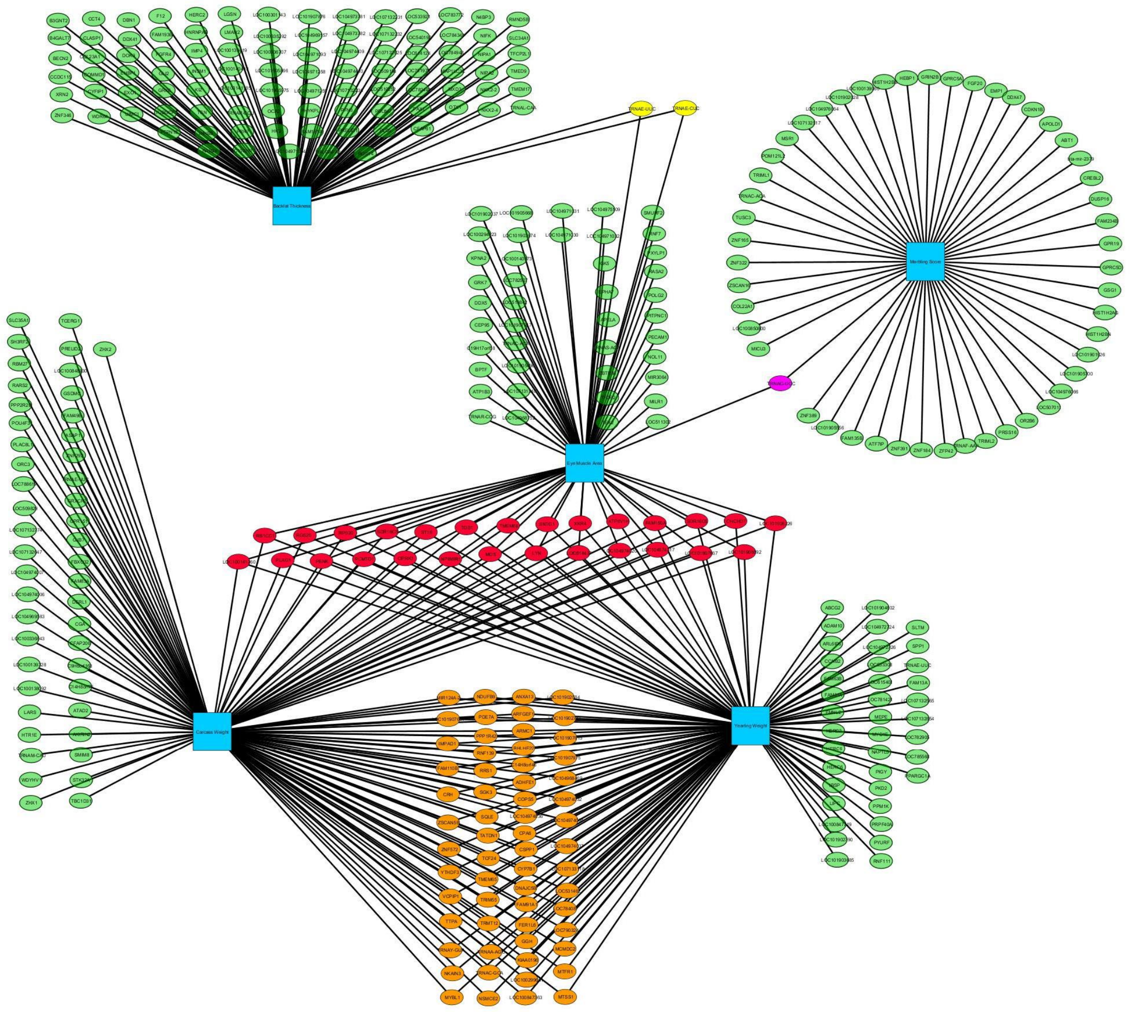

Figure S1. The global network of all related genes and traits using Cytoscape software (version 3.7.2; www.cytoscape.org). Oval nodes indicate genes and rectangle nodes indicate traits. Green nodes denote genes that are not common between traits, orange nodes denote genes that are common between carcass weight and yearling weight, red node denote genes that are common between three traits (carcass weight, yearling weight and eye muscle area), yellow nodes denote common genes between eye muscle area and backfat thickness and pink node denotes common gene between eye muscle area and marbling score traits.

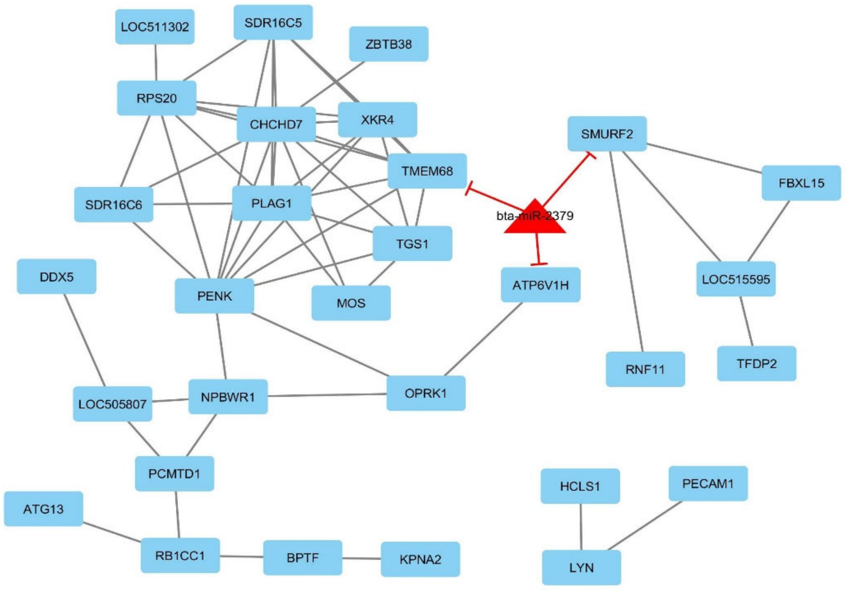

Figure S2. The network of eye muscle area related genes and miRNA using Cytoscape software (version 3.7.2; www.cytoscape.org); each node denotes a gene (rectangle) and miRNA (triangle) and edges depict miRNA-gene relationships.

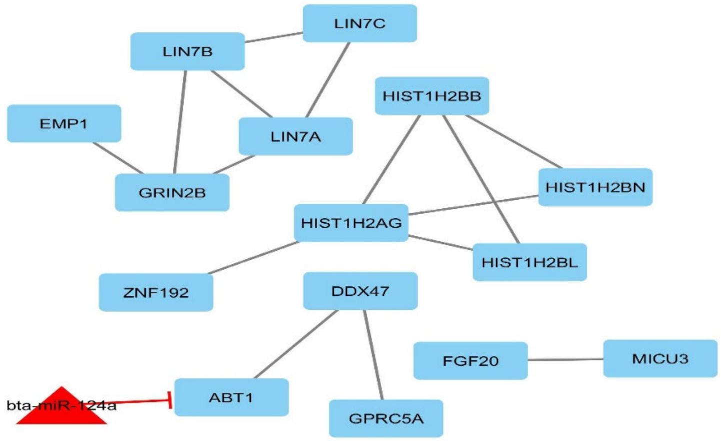

Figure S3. The network of marbling score related genes and miRNAs using Cytoscape software (version 3.7.2; www.cytoscape.org); each node denotes a gene (rectangle) and miRNA (triangle) and edges depict miRNA-gene relationships.

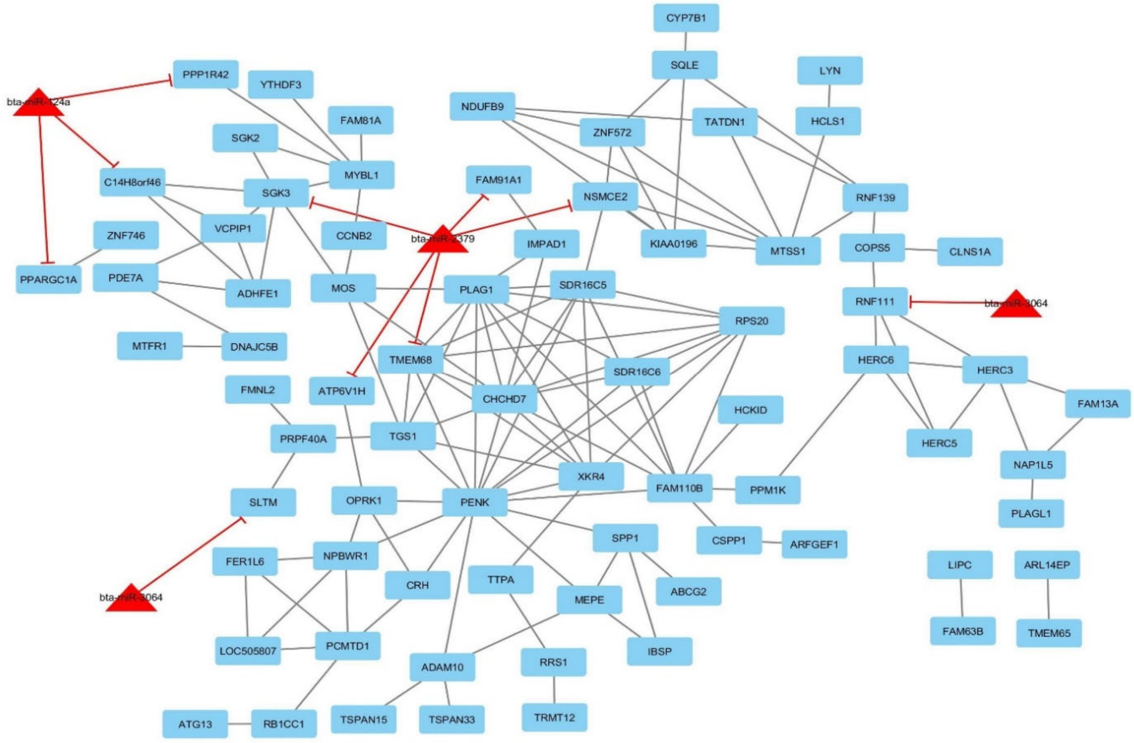

Figure S4. The network of yearling weight related genes and miRNAs using Cytoscape software (version 3.7.2; www.cytoscape.org); each node denotes a gene (rectangle) and miRNA (triangle) and edges depict miRNA-gene relationships.

© 2020 by the authors. Licensee MDPI, Basel, Switzerland. This article is an open access article distributed under the terms and conditions of the Creative Commons Attribution (CC BY) license (http://creativecommons.org/licenses/by/4.0/).
